# Dramatic response to climate change in the Southwest: Robert Whittaker's 1963 Arizona Mountain plant transect revisited

**DOI:** 10.1002/ece3.720

**Published:** 2013-08-13

**Authors:** Richard C Brusca, John F Wiens, Wallace M Meyer, Jeff Eble, Kim Franklin, Jonathan T Overpeck, Wendy Moore

**Affiliations:** 1 Department of Ecology and Evolutionary Biology, University of ArizonaTucson, Arizona, 85721; 2 Arizona-Sonora Desert MuseumTucson, Arizona, 85743; 3 Department of Biology, Pomona CollegeClaremont, California, 91711; 4 Center for Insect Science, University of ArizonaTucson, Arizona, 85721; 5 Department of Geosciences, University of ArizonaTucson, Arizona, 85718; 6 Institute of the Environment, University of ArizonaTucson, Arizona, 85718; 7 Department of Entomology, University of ArizonaTucson, Arizona, 85721

**Keywords:** climate change, elevational shifts, montane plants, Southwest

## Abstract

Models analyzing how Southwestern plant communities will respond to climate change predict that increases in temperature will lead to upward elevational shifts of montane species. We tested this hypothesis by reexamining Robert Whittaker's 1963 plant transect in the Santa Catalina Mountains of southern Arizona, finding that this process is already well underway. Our survey, five decades after Whittaker's, reveals large changes in the elevational ranges of common montane plants, while mean annual rainfall has decreased over the past 20 years, and mean annual temperatures increased 0.25°C/decade from 1949 to 2011 in the Tucson Basin. Although elevational changes in species are individualistic, significant overall upward movement of the lower elevation boundaries, and elevational range contractions, have occurred. This is the first documentation of significant upward shifts of lower elevation range boundaries in Southwestern montane plant species over decadal time, confirming that previous hypotheses are correct in their prediction that mountain communities in the Southwest will be strongly impacted by warming, and that the Southwest is already experiencing a rapid vegetation change.

## Introduction

The 7250-km (4500-mile) long North American Cordillera (or “Western Cordillera”) runs from northern Alaska to southern Mexico. This great cordillera has but one break, a low saddle between the Rocky Mountains/Colorado Plateau and the Sierra Madre Occidental (of Mexico), which forms a low-elevation, desertscrub/desert grassland biogeographic barrier between the montane biotas of temperate and tropical North America. The Sky Islands (often referred to as “Madrean Sky Islands”) are approximately 65 isolated mountain ranges that span this Cordilleran Gap in southeastern Arizona, southwestern New Mexico, and northeastern Sonora (Mexico) (Heald 1951; Marshall 1957; McLaughlin 1986, 1995; Warshall 1995; Bowers & McLaughlin 1996; Moore et al. 2013; Brusca and Moore 2013). Situated 140 km north of the U.S.–Mexico border, the Santa Catalina Mountains are one of the best known of the 32 U.S. Sky Island ranges. The vegetation of the Catalinas was first described in a classic study by Forrest Shreve (1915), and it was further elucidated through studies by Whittaker, Niering, and Charles Lowe (Lowe 1961; Whittaker and Niering 1965, 1975; Whittaker 1967; Whittaker et al. 1968; Niering and Lowe 1984; and Moore et al. 2013).

Robert H. Whittaker (1920–1980) was one of the preeminent ecologists of the twentieth century. Often described as the father of modern plant ecology, he published a series of highly influential research articles that detailed the vegetational patterns of montane regions across the United States. In 1963, Whittaker (and colleague William Niering) surveyed plants in the Santa Catalina Mountains, one of Arizona's “Sky Island” mountain ranges next to the city of Tucson. Their survey ran along the Catalina Highway, which traverses the southern slopes of the Santa Catalinas (Whittaker and Niering 1964). Their published articles refer to this as the “Catalina Highway Transect.” The Catalina Highway (aka “Mt. Lemmon Highway”), which has not changed its path since it was opened to the public in 1947, runs from 762 m (2500 ft) desertscrub in the Tucson Valley to 2791 m (9157 ft) mixed conifer forest at the top of Mt. Lemmon. Whittaker and Niering (1964) provided a detailed list of plant species for the Catalina Highway Transect, a 49-year-old data set that we use to examine changes in elevational distribution of the most common montane plants in this range.

Historical data sets, such as the Whittaker & Niering (W–N) plant data from the Catalina Highway Transect, provide enormously valuable information for examining long-term environmental trends. However, older data sets are also notoriously challenging to interpret and to use in comparative studies. W–N undertook their survey before the advent of GPS, and they did not record or map the specific site localities of their 30 plant quadrats. Instead, they randomly sampled within 1000-ft elevational bands and between the 1000-ft elevation road markers that had been established by the U.S. Forest Service along the Catalina Highway. They reported data in their published articles solely in 1000-ft elevation bands. Lacking specific site locations of the W–N quadrats, and being restricted to 1000-ft elevational bands for their plant data, limits our ability to make direct comparisons today. However, we compensate for these limitations in a number of ways: (1) we use the same approximately 20-mile stretch of the same highway, (2) we sample the area more densely, and (3) we do not recognize an elevational shift (up or down) unless it is beyond the uppermost or lowermost 1000-ft elevation band of the W–N data (see Materials and Methods). Even using this highly conservative approach, we found significant elevation shifts in most of the plants we analyzed.

The Southwest has been identified as a “global hotspot” in the CMIP5 climate change model ensemble (Diffenbaugh and Giorgi 2012), and projections from climate models suggest that temperatures in the Southwest will increase by an additional 3–6°C by the end of this century (Hayhoe et al. 2004; CLIMAS 2007, 2012). Across the region, average annual temperatures have risen over 0.84°C since 1951 (Robles and Enquist 2010), and in Arizona the average annual temperature has increased by 1.4°C since 1976 (CLIMAS 2007, 2012). Average daily temperatures in the Southwest for the 2001–2010 decade were the highest on record since 1900, and the period since 1950 has been warmer than any period of comparable length in at least 600 years (Garfin et al. 2013). Projections of whether precipitation will increase or decrease are still mixed, and the important North American Monsoon System is not well represented in most global climate models (Parmesan and Yohe 2003; Kupfer et al. 2005; Gutzler and Robbins 2010). Some regional models have suggested that precipitation in the Southwest will decrease (especially winter rainfall), but that the frequency of extreme rainfall events will increase (Diffenbaugh and Giorgi 2012; Dominguez et al. 2012). A recent analysis by Cook and Seager (2013) suggests that the onset of the Southwestern summer monsoons should shift a few weeks later in the year, although the total summer precipitation should remain about the same. However, the areal extent of drought over the Southwest during 2001–2010 was the second largest observed for any decade since 1900 (Garfin et al. 2013). Analyses using high-resolution hydrologic and climate models show that important Southwest climate-related trends, and in particular the observed regional warming, are ultimately being driven by anthropogenic increases in atmospheric greenhouse gases (Barnett et al. 2008; Bonfils et al. 2008).

Assessments of how plant communities in the Desert Southwest will respond to continued climate warming predict that increases in temperature will lead to up-elevation movement of montane species and communities (Kupfer et al. 2005; Parmesan 2006; Loarie et al. 2008). In the Madrean Sky Island region of the Southwest, this would lead to an increased area of desertscrub (western Sky Islands) or desert grassland (eastern Sky Islands), as these biomes expand upslope from the base of the mountains to replace retreating montane species, and a decrease in the area occupied by montane woodland and conifer forest as these communities move higher in elevation and/or become elevationally compressed. Models predict that upslope movement of montane plants in the Southwest should already be underway, and that upper elevation limits of montane plants may respond in mixed ways depending on changes in temperature and/or precipitation (Kupfer et al. 2005; Loarie et al. 2008; Garfin et al. 2013). However, until now there have been no direct tests of these predictions due to the lack of long-term montane plant data for the Desert Southwest. Some effects of climate change have already been noted in Arizona's Sky Island ranges in the form of changing plant phenology, increased fire, drought, pest outbreaks, and rapid spread of invasive species (Breshears et al. 2005; Kelly and Goulden 2008; Raffa et al. 2008; Van Mantgem et al. 2009; Overpeck and Udall 2010; Crimmins et al. 2010, 2011), but thus far there has been no documented movement of native montane plant upper or lower elevation boundaries. Here, we repeat Whittaker's 1963 Catalina Highway Transect to examine elevational ranges of the most common plant species 49 years later.

## Materials and Methods

Whittaker and Niering (1964) used thirty 0.1 Ha quadrats (20 × 50 m) to undertake a floral survey along the Catalina Highway Transect, from 2500 ft to 9000 ft. Data included in their 1964 study from above 9000 ft were from the Pinaleño Mountains, and data from below 2500 ft were from the Tucson Mountains. Our plant elevation comparisons focused on upland species in the 3500–9000 ft elevation range of the Catalinas. USGS topographic quadrant maps for the Santa Catalinas were first issued in 1957 (both 7.5'/1:24,000 and 15'/1:62,500), and W–N might have used those to assist in determining elevations. However, the 1000-ft elevation highway markers were also in place along the Catalina Highway when they did their sampling, and based on an examination of their original field notes, N.S.F. proposals, and rough drafts of their papers (all archived at Cornell University Library), it appears that they randomly sampled within the 1000-ft elevation bands established by these road markers; they did not record specific elevation estimates for their quadrats. Their interest was tightly focused on species ecology, not community ecology. Thus, W–N recorded and reported on a complex matrix of 110 ecological variables (e.g., habitat, plant “growth and life form,” biogeographic relationships of the species, etc.) for the individual plant species they observed. However, there is no way to determine the exact location or elevation of any of the sampling sites used in the W–N study; these were not reported in their study, and Whittaker's original, hand-written data sheets also do not record these data. W–N collected (and reported) their data in the following elevational bands for the Catalinas: 2500–3000 ft, 3000–4000 ft, 4000–5000 ft, 5000–6000 ft, 6000–7000 ft, 7000–8000 ft, and 8000–9000 ft. Here, we examine only the montane data (i.e., our plant records above 3500 ft, which is roughly the upper limit of desertscrub in the Catalina Mountains today). Thus, our plant quadrat sampling began at 3500 ft/1067 m and ended at 9111 ft/2777 m, excluding desertscrub at the base of the mountains. We have examined the Whittaker archives for the Santa Catalina Mountains at Cornell University Library's Division of Rare and Manuscript Collections and found no field notes, maps, or other material that provide additional information on the W–N quadrat locations.

Our study used the same Catalina Highway Transect as W–N, which runs from the base of the mountain to the top of Mt. Lemmon, the highest peak in the Santa Catalinas (9157 ft/2791 m). We established 33 0.1 Ha quadrats (10 × 100 m) separated from one another by an average of 50 m elevation. All plant species present in these 33 quadrats were recorded. Habitats within biomes on the southern slopes of the Catalinas are remarkably uniform (as noted by Whittaker and Niering 1964, 1965, 1975; Whittaker et al. 1968; and others), and our 0.1 Ha elongated quadrats traversed all the slope aspects and microhabitats in all the upland plant communities on the mountainside (described in Moore et al. 2013). Our sites were all farther than 0.25 km from the highway, to minimize possible road effects. The location, coordinates, plant community type, slope, aspect, and other features of all our quadrats are described in Moore et al. 2013. Variation in soil type does not appear to be an important factor regulating vegetation along the Catalina Highway Transect. Although soil types vary considerably on the northern slopes of the Catalinas, it has long been known that, due to the nature of the mountain's uplift, soil types on the southern slopes are remarkably consistent, the parent rock being Catalina gneiss with some exposed granite (Pashley 1963; Whittaker and Niering 1965, 1968a,b; Whittaker et al. 1968; Suemnicht 1977; Crittenden et al. 1980; Niering and Lowe 1984; Gehrels and Spencer 1990; Palais and Peacock 1990; Bezy 2004; Brusca and Moore 2013). We compared elevational data for the most common, upland, montane plant species (>3500 ft/1067 m) in our quadrats to the W–N upland species data. We defined “common” as a species occurring in five or more of our quadrat sites. To facilitate comparison with the W–N data, we did not sample in any areas of significant forest fire history since 1963, including the 2002 Bullock Fire or 2003 Aspen Fire. Because the W–N data are sparse for riparian/“wet canyon” sites, we limited our census to upland sites. Because the W–N data were presented in 1000-ft intervals, we use feet for elevational comparisons in this study.

**Figure 1:**
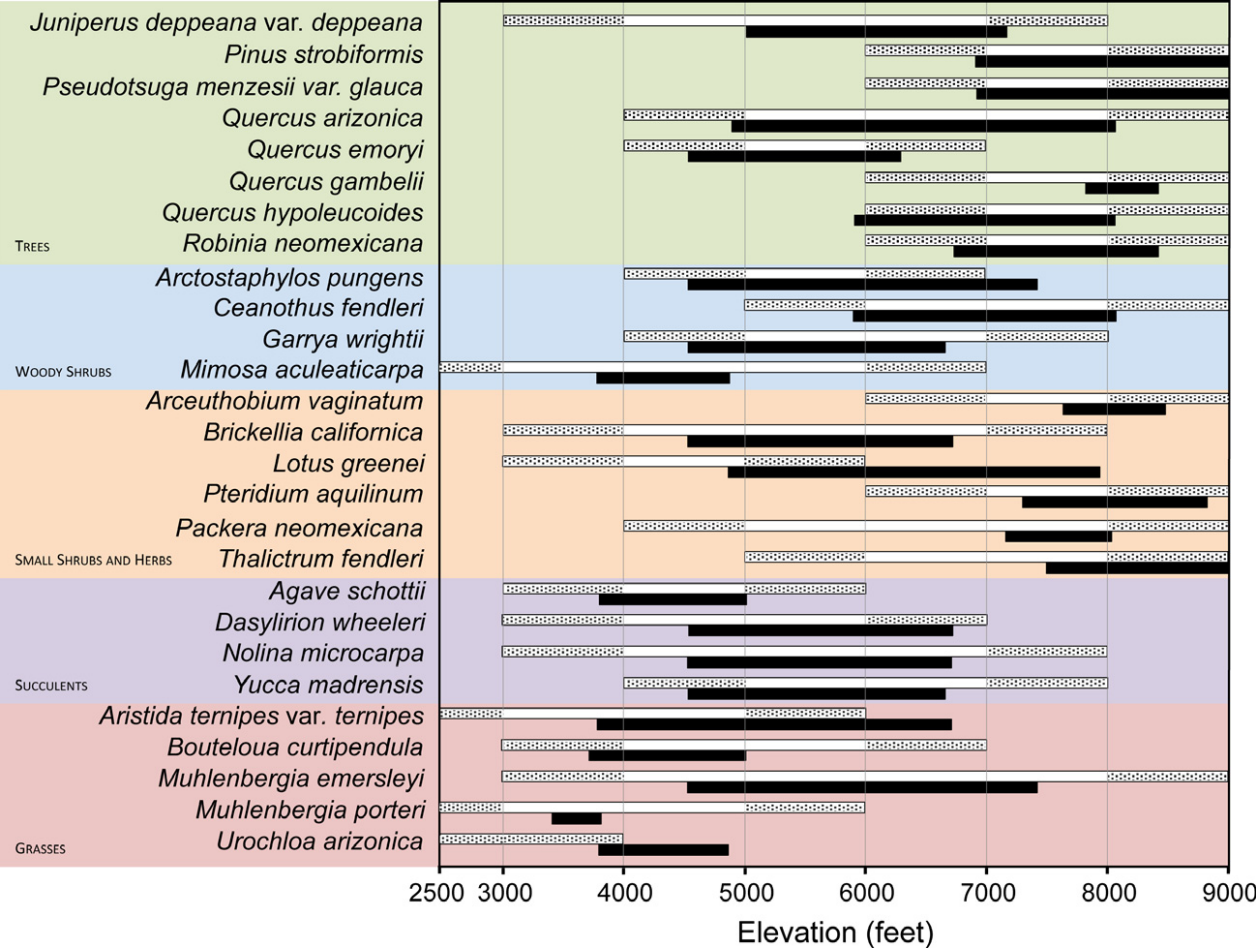
Summary of elevation range of the 27 most common upland montane plants along the Catalina Highway. White bars are 1963 elevational range data from Whittaker and Niering (1964), the two terminal (stippled) 1000-ft bands denoting Whittaker's upper- and lowermost 1000-ft vegetation bands. Black bars represent 2011 elevation data from this study. To be as conservative as possible, a change in a species elevation limit (high or low) was noted only if that species was found outside (above or below) the upper- or lowermost 1000-ft band. Thus, if anything, we underestimate the elevational change in the species since 1963 (see Materials and Methods). Following this protocol, 15 species show an unambiguous increase in lower elevation, four show an increase in upper elevation, and eight show a decrease in upper elevation.

To accommodate both the 1000-ft incremental data of W–N and our point elevation data, we plot both on a simple bar graph (Fig. 1). To be as conservative as possible, a change in a species elevation limit (upper or lower) was recorded only if our survey recorded species outside (above or below) W–N's upper- or lowermost 1000-ft bands (stippled in Fig. 1). If our data fell outside the W–N upper or lower 1000-ft band, we conservatively estimated change in distribution by recording the distance from our recorded elevation to the greatest extent of that band. Thus, our conservative approach potentially underestimates elevational change in species since 1963 (by as much as 1000 ft). For example, if the W–N data record the lower elevation boundary for a species as 4000–5000 ft, and our data record the lower elevation boundary of that species at 5100 ft, we recognize a +100 ft elevation shift, even though the actual shift could be as much as 1100 ft. Upslope changes were given positive values, and downslope movements were given negative values, allowing us to calculate changes in a species overall range by subtracting the change in a species high-elevation limit from a change in a species lower elevation limit (Table 1). Table 1 also notes the northern (i.e., temperate/Petran) versus southern (tropical/subtropical/Madrean) biogeographic relationships of the species we analyzed.

**Table 1:** Summary of 27 plant species analyzed, showing changes in lower elevation boundary, upper elevation boundary, and overall range contraction/expansion since 1963. Positive numbers indicate elevation increase (or overall range increase) and negative numbers indicate elevation decrease (or overall range contraction). Elevations are presented in feet to more easily compare them to the original Whittaker and Niering (*1*) data

	Biogeographic affinity	Elevation range (2011)	Lower elevation change (since 1963)	Upper elevation change (since 1963)	Overall elevation range contraction (since 1963)
*Juniperus deppeana* var. *deppeana* (alligator juniper)	S	5028–7162 ft (1533–2183 m)	1028	0	−1028
*Pinus strobiformis* (Southwestern white pine)	S	6962–9111 ft (2122–2777 m)	0	0	0
*Pseudotsuga menzesii* var. *glauca* (Rocky Mtn. Douglas-fir)	N	6962–9111 ft (2122–2777 m)	0	0	0
*Quercus arizonica* (Arizona white oak)	S	4893–8081 ft (1491–2463 m)	0	0	0
*Quercus emoryi* (Emory oak)	S	4539–6309 ft (1383–1923 m)	0	0	0
*Quercus gambelii* (Gambel oak)	N	7828–8412 ft (2386–2564 m)	828	0	−828
*Quercus hypoleucoides* (silverleaf oak)	S	5915–8081 ft (1803–2463 m)	−85	0	85
*Robinia neomexicana* (New Mexico locust)	N	6732–8412 ft (2052–2564 m)	0	0	
*Arctostaphylos pungens* (pointleaf manzanita)	?	4539–7434 ft (1383–2266 m)	0	434	434
*Ceanothus fendleri* (Fendler buckbrush)	N	5915–8081 ft (1803–2463 m)	0	0	0
*Garrya wrightii* (silktassle bush)	S	4539–6660 ft (1383–2030 m)	0	−340	−340
*Mimosa aculeaticarpa* (wait-a-minute bush) [=*M. biuncifera*]	S	3801–4893 ft (1159–1491 m)	801	−1107	−1908
*Arceuthobium vaginatum* (pineland dwarf mistletoe)	N	7625–8488 ft (2324–2587 m)	625	0	−625
*Brickellia californica* (California brickellia)	N	4539–6732 ft (1383–2052 m)	539	−268	−807
*Lotus greenei* (Green's lotus)	S	4893–7946 ft (1491–2422 m)	893	1946	1053
*Pteridium aquilinum* (bracken fern)	N	7297–8829 ft (2224–2691 m)	297	0	−297
*Packera neomexicana* (groundsel)[=*Senecio neomexicanus*]	N	7162–8028 ft (2183–2447 m)	2162	0	−2162
*Thalictrum fendleri* (meadow rue)	N	7477–9111 ft (2279–2777 m)	1477	0	−1477
*Agave schottii* (shindagger agave)	S	3801–5028 ft (1159–1533 m)	0	0	0
*Dasylirion wheeleri* (sotol)	S	4539–6732 ft (1383–2052 m)	539	0	−539
*Nolina microcarpa* (beargrass)	S	4539–6732 ft (1383–2052 m)	539	−268	−807
*Yucca madrensis* (mountain yucca)[=*Yucca schottii*]	S	4539–6660 ft (1383–2030 m)	0	−340	−340
*Aristida ternipes* var. *ternipes* (spidergrass)	S	3801–6732 ft (1159–2052 m)	801	732	−69
*Bouteloua curtipendula* (side-oats grama)	?	3717–5028 ft (1133–1533 m)	0	−972	−972
*Muhlenbergia emersleyi* (bullgrass)	S	4539–7434 ft (1383–2266 m)	539	−566	−1105
*Muhlenbergia porteri* (bush muhly)	S	3428–3845 ft (1045–1172 m)	428	−1165	−1593
*Urochloa arizonica* (AZ panic grass)[=*Panicum arizonicum*]	S	3806–4893 ft (1160–1491 m)	806	893	87

N = Northern biogeographic connection (i.e., temperate/Petran). S = Southern biogeographic connection (i.e., tropical/subtropical/Madrean/Tropical Deciduous Forest/Sinaloan Thorn forest). ? = Biogeographic relationship unclear.

Our plant surveys were conducted during August 5–14, 2011. The W–N surveys were made in the spring. However, the 27 species we used for our comparisons are present and easily recognized in both the spring and summer seasons in this mountain range. To further avoid possible effects of season, annuals are not included in our survey, except for *Urochloa arizonica* (Arizona panic grass), which is present and identifiable year round. Because they cannot be easily distinguished from one another, Rocky Mountain ponderosa pine (*Pinus ponderosa* var. *scopulorum*) and Arizona pine (*Pinus arizonica*) were counted together, by both W–N and in our own quadrats. In the Santa Catalinas, the Coronado National Forest manages these two trees as a “single species,” and past workers have considered Arizona pine to be a variety of ponderosa (including W–N), although this is not the current opinion. Because of this taxonomic confusion, these two species are not included in our analysis. We compared elevational data for the most common, upland, montane plant species (>3500 ft/1067 m) in our transects to the W–N 1000-ft incremental data (Table 1, Fig. 1), thus incorporating oak woodland, pine-oak woodland, pine forest, and mixed conifer forest biomes in the Santa Catalinas. One-sample *t*-tests were used to test if there were significant changes in plant elevation limits and overall elevation ranges during the 49-year period since the W–N survey. A complete list of plants by biome is given in Moore et al. (2013). Although there have been numerous taxonomic and nomenclatural changes in the plants since the W–N study, these have been addressed and there are no unresolved taxonomic issues with the species we investigated.

There are no long-term (50 year) weather data for the Santa Catalina Mountains. The most complete, local, long-term air temperature and precipitation data available since 1949 are those of the National Weather Service's weather station at the Tucson International Airport (WPO-AP) (Climsmaz 2012). Multiple diagnostic tests revealed the presence of both heteroscedasticity and first-order autocorrelation in the temperature data but not in the precipitation data. The parameters of this autoregressive model were estimated in Stata 11, and robust standard errors were used to accommodate the presence of heteroscedasticity. Precipitation data were modeled with ordinary least squares regression with the inclusion of a quadratic term to capture the curvilinear trend in the data. All analyses were conducted with Stata 11.

## Results

**Figure 2:**
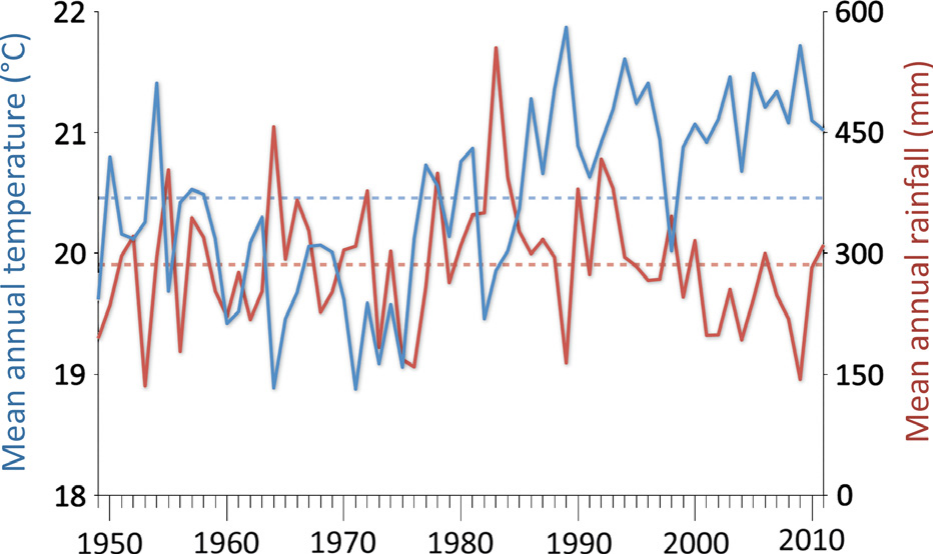
Mean annual air temperatures and rainfall in Tucson, 1949–2011. Dashed lines indicate 63-year means. Data from U.S. National Weather Service, Tucson, WPO-AP.

Figure 2 graphs National Weather Service data for mean air temperatures and mean rainfall from 1949 to 2011 in Tucson. Mean annual air temperature increased an average of 0.25°C/decade from 1949 to 2011 (*P* < 0.000). Mean annual air temperatures exceeded the 1949–2011 mean for 12 of the last 13 years. A significant curvilinear trend in mean annual precipitation was detected (*R*^2^ = 0.30, *F*_(2,60)_ = 3.36, *P* = 0.041), with precipitation decreasing over the last 20 years. In addition, mean annual rainfall has been below the 1949–2011 mean for 13 of the last 20 years, reflecting a widespread drought in the Southwest.

The 27 most common plant species in our resampling of the Catalina Highway Transect (i.e., species that occurred in five or more quadrats) are compared to the W–N transect data in Figure 1 and Table 1. Although the 1000-ft intervals of the W–N sampling limit the usefulness of their data, even at this coarse level definitive conclusions can be drawn due to the extreme changes in species' distribution. For these 27 species, the data show significant upslope elevation shifts (i.e., increases in species' lower elevation limit; *t* = 4.34, *P* < 0.001) and range contractions (*t* = −3.37 *P* = 0.002) over the 49-year period. Unambiguous upslope shifts of lower elevation boundaries for 15 (56%) species were recorded. In eight species lower elevation limits have increased by over 800 ft/245 m (Table 1). The overall elevational ranges of 16 species (59%) have been compressed (Table 1). Changes in the upper elevation limits of species occurrence are mixed (*t* = −0.22, *P* = 0.83), with some species having shifted up (four), some down (eight), and others showing no change (15).

## Discussion

Robert Whittaker's classic 1963 Catalina Highway transect (W–N 1964) allows for a unique, 49-year comparison, during which time Southwest temperatures have increased significantly (an average of 0.25°C/decade in Tucson). This is the longest documented timeframe for direct comparison of montane plant elevational boundaries in North America, spanning 5 decades of warming climate in the Southwest. Our comparison revealed that, as models predict, 15 of the 27 most common plant species in the Catalinas have increased their lower elevational boundary, whereas four have increased their uppermost elevational boundary, and eight have decreased their upper elevation boundary.

**Figure 3:**
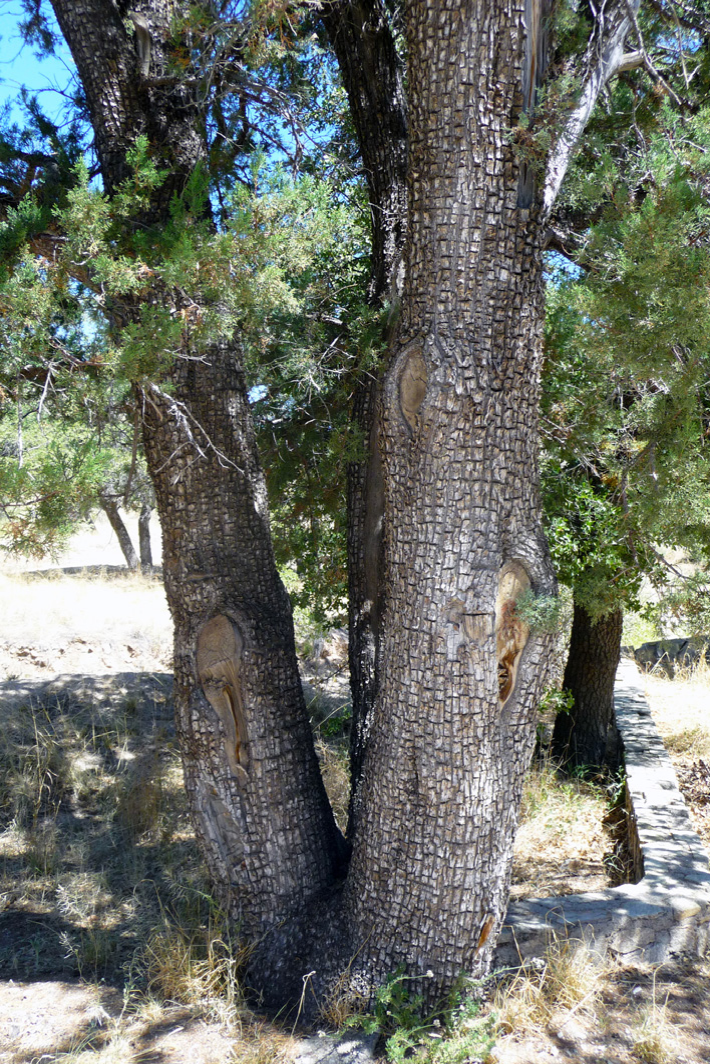
Today, the first alligator junipers (*Juniperus deppeana* var. *deppeana*) along the upland slopes of the Catalina Highway make their appearance at approximately 5000 ft. Fifty years ago, Whittaker and Niering (1964) reported this species from desert and desert grassland at 3000 to 4000 ft.

It is worth noting that even a casual observer could recognize the changes in plant elevation boundaries since the W–N surveys of 50 years ago – without so much as stopping the car as they drive up the Catalina Highway. Large and conspicuous plants such as alligator juniper, bracken fern, beargrass, and sotol simply do not begin to appear in the Catalinas until much higher elevations than reported by W–N in 1964. For example, W-N reported the lower elevation of alligator juniper from “mesic desert and desert grassland” in their 3000–4000 ft sampling belt, which would put it at places like the Babad Do'ag Trail and Molino Canyon Overlook along the Catalina Highway (20 years later, Niering and Lowe, 1984, also recorded this species from 3500 ft, the elevation of Babad Do'ag Vista). Today, however, the first alligator junipers make their appearance at around 5000 ft, or a few hundred feet lower in moist/riparian areas (Fig. 3). David Bertelsen has been conducting a plant phenology study along a 4158 ft-elevation segment of the Finger Rock Trail, on the southern slopes of the Catalinas, for over 25 years (see Crimmins et al. 2010, and cited therein). Bertelsen's records of alligator juniper (5000–7255 ft) match our Catalina Highway Transect almost perfectly (D. Bertelsen, pers. comm.). Bracken fern, a member of moist soil pine forest communities, today begins to appear just below the Catalina Mountain Visitor Center, approximately 7500 ft. W–N recorded it from 6000 to 7000 ft, which would put it at Middle Bear Picnic Grounds and Windy Point, which, today, are dry mixed chaparral and Madrean pine-oak woodland habitats. Today, beargrass makes its first appearance in Molino Basin (approximately 4750 ft); W–N recorded it from 3000 to 4000 ft, which would put it around Babad Do'ag Vista (today, a desertscrub habitat). Today, sotol does not occur much lower than 4100 ft, and is first seen along the highway where it intersects Molino Canyon; the W–N records from 3000 to 4000 ft indicate that sotol occurred at and below the elevation of Babad Do'ag Vista, which today is desertscrub habitat.

It has long been predicted (and observed) that plants respond individually to stressors such as climate warming (e.g., Whittaker 1967; Chen et al. 2011; Schwilk and Keeley 2012; Fleishman et al. 2013), especially at their upper elevational boundaries, and that appears to be the case in our study. Even if mean annual rainfall remained unchanged over time, both its increasing variability and shift in timing, and the influence of rising air temperatures can be expected to influence water balance in plants by way of atmospheric evaporative demand (Kimball et al. 2010; Robles and Enquist 2010; Taiz and Zeigler 2010; Williams et al. 2011, 2012). Of the eight plant species that have lowered their uppermost elevation boundary in the Santa Catalina Mountains since 1963, six are subtropical Mexican species whose northernmost range limit is in Arizona's Sky Islands: Wright silktassle (*Garrya wrightii*), wait-a-minute (*Mimosa aculeaticarpa*), Beargrass (*Nolina microcarpa*), mountain yucca (*Yucca madrensis*), bullgrass (*Muhlenbergia emersleyi*), and bush muhly (*Muhlenbergia porteri*). All these are summer monsoon–dependent species. Average summer-fall evaporative demand has been increasing steadily in recent decades of atmospheric warming, and it has been the highest on record since 2000 (Williams et al. 2012). Recent research documents that summer-fall atmospheric evaporative demand is just as important as winter precipitation in stressing montane plants, and that this available water deficit has impacted Southwestern forests for centuries during periods of warming and/or drought (Williams et al. 2011, 2012). In fact, climate model projections of winter precipitation and summer-fall evaporative demand suggests that megadrought-type forest drought-stress conditions will exceed those of the megadroughts of the 1200s and 1500s on a regular basis by the 2050s, and that this condition has prevailed over about 30% of the past 13 years in the Southwest (Williams et al. 2012). We hypothesize that this increasing summer-fall atmospheric evaporative demand (driven by rising air temperatures), coupled with decreasing mean annual precipitation over the past 20 years, has led to drought-like conditions that have impacted these summer monsoon–adapted plant species at the northern fringe of their geographic distribution. Of the four species that have increased their upper elevation boundary since 1963, none is strictly tropical/southern species.

Although no species showed a significant downward shift in their lower elevation, silverleaf oak (*Q. hypoleucoides*) was recorded 85 ft (26 m) lower than recorded by the W–N survey; however, this is below the resolution of our study. This oak is a subtropical, Madrean, eurytopic species that is one of the most widespread in the Santa Catalina Mountains, and across most of the Madrean Sky Islands, and it appears to be extending its range northward from Mexico. In the Catalinas, it spans an elevational range of over 2160 feet. Overall, plant species appear to have adjusted their upper and lower elevational limits individually, as predicted by Whittaker (1967) and others (Crimmins et al. 2010; Chen et al. 2011; Schwilk and Keeley 2012), in contrast to other predictions suggesting that increases in the lower elevation boundary will “follow along behind” increases in the upper elevation boundary (Peters and Lovejoy 1992). Individual plant species responses to warming climates also are well documented for the Wisconsin/Holocene transition (see below), and are also documented for animal species (Wilson et al. 2007; Chen et al. 2009), both leading to changes in community composition.

Recent phenological studies in the Santa Catalina Mountains support our observations of plant community changes on the south slope of this range. Over the past 20 years, low-elevation (desert) annuals have shifted their germination times to later in the year, due to delays in the occurrence of winter rains, which now peak in December rather than October. This shift in the timing of rainfall has led to an increase in abundance of cold-adapted plant species in that they are able to germinate successfully in cooler conditions (Kimball et al. 2010). This same phenomenon could be affecting the higher elevation montane plants in our study and impacting their elevational occurrence. Similarly, a 20-year data set across a 1200-m elevation gradient starting at the base (desertscrub) of the Catalinas revealed that 25.6% of the plant species showed a significant shift upward in the lowest elevation at which they flowered (Crimmins et al. 2009). Onset of flowering in summer is strongly linked to the amount and timing of the onset of the summer monsoon rains (Crimmins et al. 2011).

Chen et al. (2011) used a meta-analysis approach, estimating a mean shift of 12.2 m uphill per decade for plants and animals worldwide, noting that individual species vary greatly in their rates of change and suggesting that the range shift of each species depends on multiple species traits as well as multispecies interactions. They also found that taxonomic affinity is not a consistent predictor of response rates. The data from our study agree with these findings. The mean lower elevation boundary of the 27 montane plants in our study is conservatively estimated to have risen at a mean rate of 27.6 m/decade, although this figure is somewhat abstract because we compare only two points in time, 49 years apart. However, these data suggest that the Southwest is experiencing more rapid change in montane plant communities than most other regions of the world, as predicted by climate models.

Global mean land surface temperature has warmed 0.27°C/decade since 1979 (IPPC 2007), and the effects of this rapid climate change on plant distributions has been a subject of growing interest worldwide (McCarty 2001; Parmesan and Yohe 2003; Kupfer et al. 2005; Rosenzweig et al. 2007; Kelly and Goulden 2008; Normand et al. 2009; Engler et al. 2011; Williams et al. 2011, 2012). Most work on plant distributional responses to climate change has been model based. However, declines in Arctic-Alpine plants at their southern margins of distribution were reported from the Rocky Mountains in Montana (Lesica and McCune 2004), and widespread conifer forest dieback in the western United States has been attributed to droughts and bark beetle outbreaks mediated by warmer air temperatures and/or drought (e.g., Breshears et al. 2005; Raffa et al. 2008; Van Mantgem et al. 2009; Fellows and Goulden 2012). Studies on plant phenology have attributed longer growing seasons and early onset of flowering to climate warming (Parmesan 2006; Crimmins et al. 2009, 2010), as well as widespread changes in plant growth (Harrison et al. 2010; Williams et al. 2012). Most field studies that have shown evidence of plant shifts have focused on the edges of their geographical ranges (Grabherr et al. 1994; Sturm et al. 2001; Cannone et al. 2007; Lenoir et al. 2008). There are only a few published tests for elevational shifts of montane plants based on decadal field data (see Lenoir et al. 2008 and Chen et al. 2011 and cited work for Europe, and Kelly and Goulden 2008 for California); however, none of these studies documented changes in lower elevation boundaries or elevational ranges on mountains in the Southwest, and our study is the first to do so.

In 1994 an international effort established the GLORIA “master site” (Global Observation Research Initiative in Alpine Environments) – an array of approximately 1100 1 m × 1 m permanent plots at the alpine-nival ecotone (between 2900 and 3450 m) in the Tyrolean Alps, Austria. The alpine-nival ecotone is the transition between the lower alpine grassland/tundra zone (dominated by *Carex curvula* and *Oreochloa disticha*) and the upper sparsely vegetated nival zone (open scree and rock dominated by cushion plants, small rosettes, and graminoids). Pauli et al. (2007) reported on a resurvey of the site after 10 years, noting that most plants had moved higher on the mountain (both their lower and upper elevational range end points had moved upslope). A similar upslope movement of subalpine forests (by 60–80 m) was noted in the Polar Urals over the last 35 years (Moiseev and Shiyatov 2003). Similarly, Grabherr et al. (2001) used 50- to 80-year-old historical, high-elevation (alpine) plant records from the Alps to show that some species had migrated upslope, including the Alpine sedge *Carex curvula*, a dominant plant of Alpine meadows, which had shifted its lower elevation boundary from approximately 2800 to 3468 m.

The Wieslander Project, a joint initiative of the University of California at Berkeley and Davis, is digitizing photographs, maps, and vegetation plots from a large portion of California dating from 1928 to 1940, and comparing these data to contemporary landscapes. (Kelly et al. 2005; Keeler-Wolf 2007). The project is mapping changes in landscape vegetation, primarily due to logging, fire, urbanization, and land-use conversion. However, in the Sierra Nevada they found evidence of an upslope shift in the ponderosa pine belt, which they suggest is due to climate change and increasing length of summer droughts with resulting high mortality among recruiting ponderosa pine seedlings (Thorne et al. 2006), as predicted by the Williams et al. (2012) analysis. In a study somewhat similar to ours, Kelly and Goulden (2008) reexamined the “Deep Canyon Transect” in the Santa Rosa Mountains of Southern California (also see Zabriskie 1979; Breshears et al. 2008; and Schwilk and Keeley 2012) and found an upslope shift in the mean elevation of 10 plant species based on weighted cover. However, the Kelly & Goulden study did not assess actual species elevation ranges (as we do in our study), and it did not document lower and upper elevational boundaries for the species in question.

It has long been known from the paleontological record (mainly from packrat middens and lake sediment cores) that plants in the Southwest have moved downslope/upslope during cooler/warmer periods, as well as migrating latitudinally on an individualistic basis. During the last glacial episode (the Wisconsin), the area we recognize today as Sonoran Desert was much cooler and supported not desertscrub but chaparral plants, pinyon pines, junipers, and oaks. These Wisconsin woodlands were characterized by singleleaf pinyon (*Pinus monophylla*), junipers (*Juniperus* spp.), scrub oak (*Quercus turbinella*), and Joshua tree (*Yucca brevifolia*) (Martin 1963; Betancourt et al. 1990, 1991; Anderson and Van Devender 1991; Davis 1995, 1999; MacAuliffe and Van Devender 1998; Thompson and Anderson 2000; Lozano-García et al. 2002; Van Devender 2002; Holmgren et al. 2003, 2011; Lyford et al. 2003). Late Wisconsin-dated packrat middens from 5100 ft in the Catalina Mountains (Pontatoc Ridge) document woodlands of Arizona cypress (*Cupressus arizonica*), Douglas-fir (*Pseudotsuga menzesii* var. *glauca*), hybrid singleleaf/Colorado (two-needle) pinyon (*P. monophylla/P. edulis*), border pinyon (*Pinus cembroides* var. *bicolor*), and Rocky Mountain ponderosa pine (Van Devender 1977). During the Wisconsin, singleleaf pinyon was so common that it probably grew almost everywhere pines occurred, in what is now the Sonoran Desert Region. Today, however, singleleaf pinyon is rarely seen south of the Mogollon Rim in Arizona, and Joshua trees are restricted to a small refugial area near the California–Arizona border. Neither of these latter two species found refuge in the higher elevations of the Sky Islands (although many other species did), but instead retreated northward as climates warmed. In Arizona today, the Colorado pinyon is also found almost exclusively north of the Mogollon Rim, although refugial patches do occur in some high-elevation localities in the Chiricahua Mountains (Brusca and Moore 2013; SEINet). With the onset of Holocene warming, the pinyon–juniper–oak landscape of the Sky Island Region's valleys began to move northward and up mountain slopes, to be gradually replaced by desertscrub and grassland from the south. By about 11,000 years ago, singleleaf pinyon, Colorado pinyon, and other temperate species had vacated the lowlands. The Sonoran Desert plants we see today began arriving (from the region of Sonora and Sinaloa) in the Sky Island Region approximately 11,000 years ago, although some, such as ironwood (*Olneya tesota)* and organ pipe cactus (*Stenocereus thurberi*), probably did not get into this region until about 4000 years ago (MacAuliffe & Van Devender 1998; Thompson and Anderson 2000; Van Devender 2002).

Overall, these studies indicate that montane plant communities throughout the Northern Hemisphere are moving higher in elevation in step with climate warming, a pattern seen in our study in the Santa Catalina Mountains of southern Arizona. The shifts in plant ranges we observed in the Santa Catalina Mountains indicate that the area occupied by montane woodland and conifer forests in the Desert Southwest is likely to decrease even more with predicted increases in temperature, and that regional plant community composition has and will continue to change with further warming as plant species respond individualistically to changing climates.
