# Dramatic response to climate change in the Southwest: Robert Whittaker's 1963 Arizona Mountain plant transect revisited

**DOI:** 10.1002/ece3.832

**Published:** 2013-09-19

Richard C. Brusca, John F. Wiens, Wallace M. Meyer, Jeff Eble, Kim Franklin, Jonathan T. Overpeck &Wendy Moore

Ecology and Evolution 2013; 3(10): 3307–3319

doi: 10.1002/ece3.720

Just after our paper appeared in Ecology & Evolution's “Early View” online, we discovered an error in how we reported the original Whittaker and Niering (1964) sample data. We reported that they used 30 0.1-Ha upland (nonriparian/nonwet canyon) samples between 3500 ft and 9000 ft along the Catalina Highway. However, these 30 samples were actually what Whittaker called “grouped samples” as described by Whittaker (1967) (p. 212). Each “grouped sample” is actually combined data from either 5- or 10 0.1-Ha quadrats. These are the 30 sample points in Figure 1 (Whittaker and Niering 1964), labeled *b* through *f*, between 3500 ft and 9000 ft.

While this discovery reveals differences in sampling efforts, it does not change the results of our statistical analysis (one-sample *t*-test) or our conclusions, and we are confident that our sampling effort was sufficient. As noted in our paper, soil types on the southern slopes of the Catalinas, especially along the Catalina Highway, are remarkably uniform, and plant species presence is highly predictable based on elevation and aspect, especially for the species that we analyzed. It is possible that if a given species was normally distributed across elevation gradients, it would be easier to miss that species’ occurrence when sampling the tails of its distribution (i.e., the lowest and highest elevations). However, our samples were separated from one another (on average) by just 50 m in elevation, and species showing upslope movement of lower elevation had an average gain of nearly 260 m (850 ft) – more than five times our elevation sampling breaks – indicating that our sampling effort was adequate. 
